# A reappraisal of *ASXL1* mutation sites and the cohesin-binding motif in myeloid disease

**DOI:** 10.1038/s41408-023-00876-w

**Published:** 2023-06-26

**Authors:** Steven M. Johnson, James Haberberger, Jonathan Galeotti, Lori Ramkissoon, Catherine C. Coombs, Daniel R. Richardson, Matthew C. Foster, Daniel Duncan, Joshua F. Zeidner, Naomi L. Ferguson, Nathan D. Montgomery

**Affiliations:** 1 Department of Pathology and Laboratory Medicine, The University of North Carolina at Chapel Hill School of Medicine, Chapel Hill, NC USA; 2 Foundation Medicine, Inc, Cambridge, MA USA; 3 Division of Hematology, Department of Medicine, The University of North Carolina at Chapel Hill School of Medicine, Chapel Hill, NC USA; 4 Lineberger Comprehensive Cancer Center, University of North Carolina, Chapel Hill, NC USA; 5 Present Address: UC Irvine, 1001 Health Sciences Road, Irvine, CA 92697 USA; 6 Present Address: Novartis Pharmaceuticals, Cambridge, MA 02139 USA; 7 Present Address: GRAIL, Inc., 4001 E NC 54 Hwy Assembly Suite 1100, Durham, NC 27709 USA; 8 Present Address: Tempus Labs, Inc., 25 Alexandria Way, Durham, NC 27703 USA

**Keywords:** Haematological cancer, Haematological cancer

Dear Editor,

Emerging evidence supports that *ASXL1* mutation in myeloid neoplasia leads to aberrant protein gain-of-function rather than loss-of-function as initially thought [1]. Thus, a reassessment of the potential biologic relevance of the site of mutation in *ASXL1* in patients with myeloid disease is warranted. To date, a large-scale comparison of patients with the c.1934dupG (p.G646Xfs*12) hotspot mutation vs. those with other *ASXL1* mutations has not been performed. In addition, *ASXL1* has a role in stabilizing the cohesin complex by means of a cohesin-binding motif (CBM) [2], and patients with CBM mutations have been insufficiently characterized. Thus, we sought to further characterize the clinicopathologic and genetic features of patients by *ASXL1* mutation site using a large clinical dataset.

We retrospectively analyzed a cohort of 6,043 adults with a documented or suspected myeloid neoplasm and at least one mutation identified by FoundationOne®Heme testing between January 1, 2014, and August 15, 2021. The gene panel and coverage (Supplementary Table 1), as well as the sequencing methods (Supplementary Methods), have been previously published [3, 4]. The research-consented patient database was queried for specimens assigned a diagnosis category of acute myeloid leukemia (AML), myelodysplastic syndrome (MDS), myeloproliferative neoplasm (MPN), or MDS/MPN overlap. Logistic regression analysis confirmed significant genetic differences between AML, MDS, MPN, and MDS/MPN categories typical of each myeloid phenotype [5], supporting separation into these four groups for study purposes (Supplementary Fig. 1).

Pathogenic *ASXL1* mutations were identified in 1,414 patients, occurring in 18% of AML and 26% of chronic myeloid neoplasms (Supplementary Table 2), and most occurred in the final exon (Fig. 1A). Twenty-eight (2.0%) patients had multiple *ASXL1* mutations, and *ASXL1* was the sole mutated gene in only 52 patients (3.7%). The most common *ASXL1* mutation was c.1934dupG (p.G646Xfs*12), and this was the sole or dominant *ASXL1* mutation in 520 cases (37%, collectively referred to as *ASXL1*^c.1934dupG^ hereafter, Supplementary Fig. 2). The remaining 894 patients (63%) had mutations at other sites in the *ASXL1* gene (*ASXL1*^other^).

**Fig. 1:**
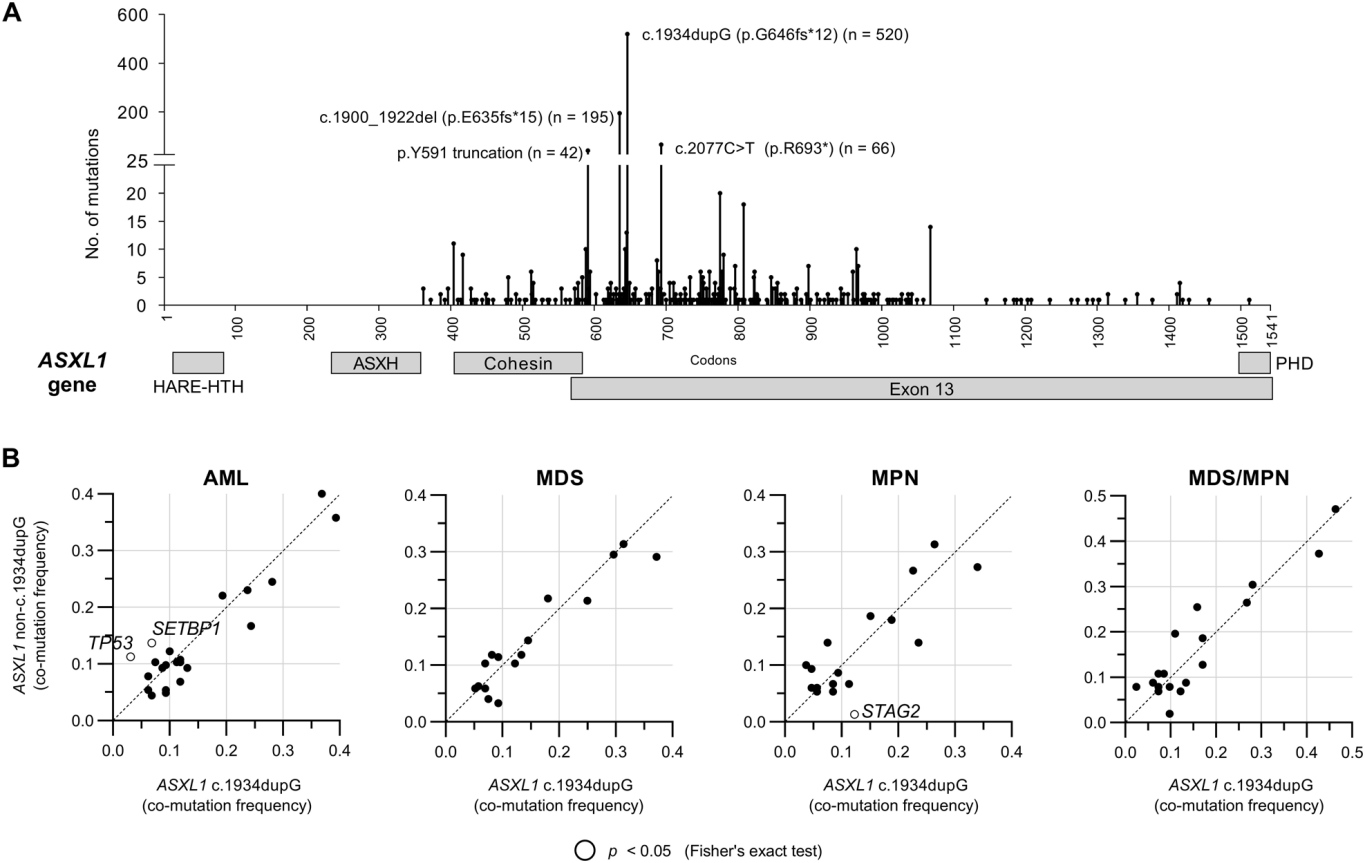
Mapping of *ASXL1* mutations. **A** Lollipop plot of *ASXL1* mutations by codon sites in all myeloid neoplasms (*n* = 1414). **B** Frequencies of ASXL1c^.1934dupG^ and *ASXL1*^other^ co-mutation with individual genes by phenotype (genes mutated in ≥5% of cases are shown). Gene names with frequency differences with a *p*-value < 0.05 are shown. *ASXL1* Ensembl gene IDENSG00000171456.12; Ensembl transcript ENST00000375687.4 HARE-HTH HB1, ASXL, restriction endonuclease helix-turn-helix domain (codons 11–82), ASXH ASX homology domain (codons 237–361), Cohesin cohesin-binding motif (codons 401–587), Exon 13 codons 573–1541, PHD plant homeodomain zinc finger (codons 1500–39).

There were no significant differences in age, sex, or genomic ancestry between *ASXL1*^c.1934dupG^ and *ASXL1*^other^. We noted slightly fewer *ASXL1*^c.1934dupG^ mutations in patients with MDS (*ASXL1*^c.1934dupG^:*ASXL1*^other^ 0.48:1) compared to AML (0.65:1, *p* = 0.03) and MPN (0.60:1, *p* = 0.01) and those in whom *ASXL1* was the sole mutation (Supplementary Fig. 3A). However, these trends may be due to our VAF-based reporting thresholds (see Supplementary Methods), as *ASXL1* VAFs were lower in singly mutated patients and those with MDS (Supplementary Fig. 3B). Across all *ASXL1*-mutated patients, *STAG2* mutations were more likely to be seen with *ASXL1*^c.1934dupG^ (21% vs. 16%, *p* = 0.02), whereas *SETBP1* mutations were more commonly co-mutated with *ASXL1*^other^ (15% vs. 10%, *p* = 0.01).

More prominent differences emerged within phenotypic subsets (Fig. 1B). For instance, *STAG2* mutations were strongly associated with *ASXL1*^c.1934dupG^ in MPN, with an *ASXL1*^c.1934dupG^:*ASXL1*^other^ ratio of 9:1 (*p* < 0.01). In contrast, two genes in AML, *TP53* and *SETBP1*, had a significantly higher co-mutation rate with *ASXL1*^other^ (*TP53*: 11% vs. 3% in *ASXL1*^c.1934dupG^, *p* < 0.01; *SETBP1*: 14% vs. 7%, *p* = 0.04). As such, we sought to determine whether other specific *ASXL1* mutations were associated with *TP53* or *SETBP1* co-mutation in AML (Supplementary Fig. 4). Mutations at codon 693 were significantly more frequent in cases of *ASXL1*^mut^*TP53*^mut^ AML compared with *ASXL1*^mut^*TP53*^wt^ AML (22% vs. 3.5%, respectively, *p* < 0.01). Similarly, the p.R404* variant was more often seen in *ASXL1*^mut^*SETBP1*^mut^ AML (7.3% vs. 0.05% of *ASXL1*^mut^*SETBP1*^wt^ AML, p = 0.01), though the total number of mutations in each group was small (*n* = 3, *n* = 2, respectively). These results confirmed that the c.1934dupG variant occurs in a similar patient population to other *ASXL1* variants. However, subset analysis supported that *ASXL1*^c.1934dupG^ and *ASXL1*^other^ may be associated with some phenotypic and co-mutational tendencies.

The ASXL1 protein directly interacts with core cohesin proteins between amino acids 401 and 587 [2], a region denoted the cohesin-binding motif (CBM) [6]. Thus, we also sought to characterize *ASXL1* CBM mutations to investigate for potential relationships to cohesin gene mutations (Supplementary Fig. 5). Overall, only seven patients with CBM mutations had cohesin co-mutations (Fig. 2A). Mutations consisted of either *STAG2* (*n* = 6) or *RAD21* (*n* = 1) and occurred in all disease phenotypes. VAF data suggested cohesin mutations were subclonal to CBM mutations in three cases. Notably, in 5 of 6 CBM/*STAG2* co-mutated cases, the CBM mutation clustered within a 21-codon region between amino acids 491 and 512.

**Fig. 2:**
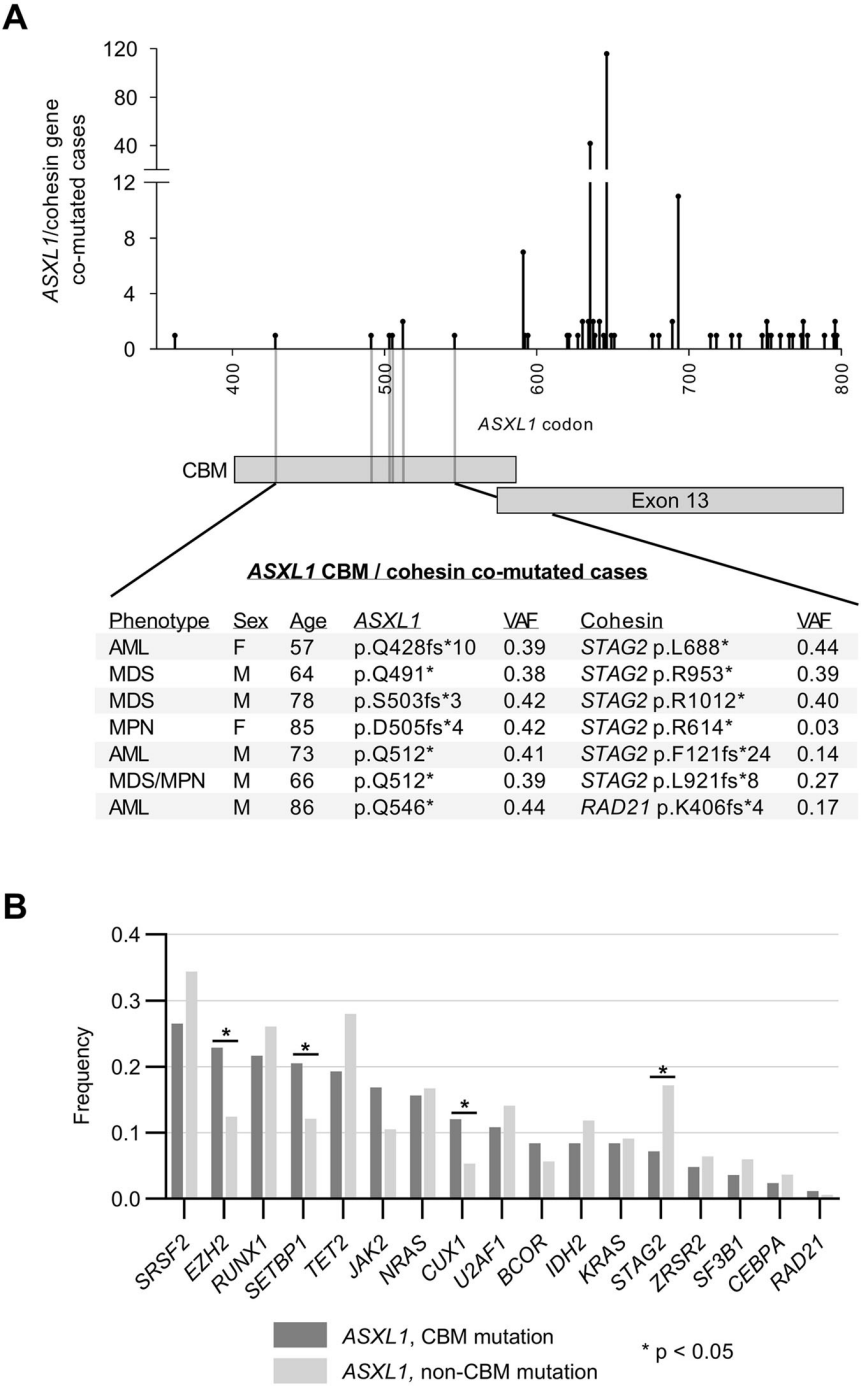
*ASXL1* cohesin-binding motif mutations. **A** Lollipop plot of *ASXL1* mutations in all patients with cohesin gene co-mutations within and just beyond *ASXL1*’s cohesin-binding motif (CBM). **B** Frequencies of co-mutation with individual genes in those with CBM and non-CBM *ASXL1* mutations. VAF variant allele fractions. *STAG2* VAFs in males were halved given this gene’s location on the X chromosome.

The overall frequency of CBM mutations in cohesin-mutated patients (7/435, 1.6%) was significantly lower than in cohesin-wild type patients (76/979, 7.8%, *p* < 0.01). When restricting analysis to the *ASXL1*^mut^ cohort, the proportion of CBM mutations was also lower in cohesin co-mutated cases (*n* = 7/244, 2.9%) than in *ASXL1*-mutated patients overall (*n* = 84/1414, 5.9%, *p* = 0.06). In these patients, cohesin mutations became more frequent just beyond the CBM, as we found that the frequency of cohesin co-mutation in the 7-codon region after the CBM (15%) was similar to the rate found throughout the remainder of the *ASXL1* gene (18%). This further supported some degree of exclusivity between cohesin and CBM mutations. Lastly, we investigated *ASXL1*^mut^ patients for differences in co-mutation frequencies of non-cohesin genes based on CBM mutation status (Fig. 2B) and found that cases with CBM mutations were significantly enriched for *SETBP1*, *EZH2*, or *CUX1* co-mutation (each *p* < 0.05).

To our knowledge, our cohort represents the largest number of patients (*n* = 1414) with *ASXL1*-mutated myeloid disease analyzed to date. A reappraisal of the spectrum of *ASXL1* mutation sites is indicated given the recent discovery that *ASXL1* mutations result in a stable, truncated gain-of-function protein, which promotes leukemogenesis via deregulation of BAP1 [1], a key component of the polycomb repressive deubiquinase complex [7]. It also appears that *ASXL1* mutation site has effects on downstream epigenetic changes. For instance, Binder et al. identified heterogeneous expression profiles in chronic myelomonocytic leukemia patients with different *ASXL1* mutations [8]. In their study, the two patients with the most distal *ASXL1* truncation sites at codons 695 and 957 had gene expression profiles intermediate between those patients with the c.1934dupG mutation and those who were *ASXL1*-wild type [8].

Few patients in our cohort had biallelic *ASXL1* mutations, which is consistent with the gain-of-function mechanism resulting from *ASXL1* mutation. Our data also confirmed the c.1934dupG occurs in a patient population with similar demographic and clinicopathologic features as non-c.1934dupG mutations, further supporting its pathogenicity in myeloid disease [9, 10]. However, in AML, patients with non-c.1934dupG *ASXL1* mutations more frequently harbored *TP53* and *SETBP1* mutations, both of which are associated with adverse-risk in myeloid disease. It currently remains unclear if *ASXL1* mutation site has an effect on patient outcomes. While in AML, patient outcomes appear similar between those with c.1934dupG mutations and non-c.1934dupG mutations [11], in primary myelofibrosis, c.1934dupG mutation is associated with worse overall survival [12, 13]. Notably, we found a very strong predilection for *STAG2* co-mutation in c.1934dupG-mutated MPNs. Overall, results of our mutation site analysis raise the possibility that site of *ASXL1* truncation may confer selective pressures for acquisition of different driver mutations. Thus, future study is warranted to investigate for potential prognostic impacts based on *ASXL1* mutation site, and specifically with the presence of *TP53, SETBP1*, and *STAG2* co-mutations.

While *ASXL1*’s role in chromatin modification is well-established, the gene also appears to function in cohesin complex stabilization. Li et al. [2] demonstrated direct protein-protein interactions between a cohesin-binding motif (CBM) in the ASXL1 protein and the gene products of *RAD21, SMC1A, SMC3*, and *STAG2*. Scarpa et al. subsequently analyzed a large AML cohort and found that, of 18 patients with an *ASXL1* CBM mutation, none had a mutation in any cohesin gene [6]. In our study, CBM mutations were generally—but not entirely—mutually exclusive with cohesin mutations, occurring at a significantly lower rate than in cohesin-wild type patients. Independent of cohesin mutation status, we found that *EZH2*, *SETBP1*, and *CUX1* mutations were significantly more likely to co-occur with CBM mutations than non-CBM *ASXL1* mutations. These collective findings suggest cohesin co-mutations are selected against in patients with CBM mutations and that alternative non-cohesin mutation pathways may drive leukemogenesis when the CBM is compromised. Further investigation into the biologic implication of CBM mutations is warranted, however, as it is unknown if alterations in the CBM—which occurs *before* the final exon—result in protein loss-of-function or gain-of-function.

Important limitations in our study were the lack of available comprehensive data and rigorously defined pathologic diagnoses for the cohort. Thus, it is inevitable that some patients would not meet strict World Health Organization diagnostic criteria and that our diagnosis categories may be more clinically heterogeneous. Thus, while our results may not be representative of all patients with myeloid disease, this cohort nonetheless allowed for the unique opportunity to explore the genetics of a large number of patients with *ASXL1* mutation.

In summary, our data from a large clinical cohort suggests that *ASXL1* mutation site may be biologically relevant in patients with myeloid disease. As targeted treatment options emerge for patients with *ASXL1* [14] and cohesin [15] mutations, further study is warranted to assess for potential therapeutic and prognostic implications in patients with these co-mutations.

## Supplementary information

Supplementary Material

## Data Availability

The datasets generated during and/or analyzed during the current study are available from the corresponding author on reasonable request.
