# Supplementary material for: A reappraisal of ASXL1 mutation sites and the cohesin-binding motif in myeloid disease
Source: Blood Cancer J. 2023 Jun 26;13(1):96. doi: 10.1038/s41408-023-00876-w (PMC10293251; doi:10.1038/s41408-023-00876-w)
Supplement: Supplementary file 1 — Supplementary Material [file 41408_2023_876_MOESM1_ESM.docx]

Supplementary Material: Table of Contents

[Methods 2](#_Toc135312912)

[Table 1. Gene panel 4](#_Toc135312913)

[Table 2. Patient cohort 6](#_Toc135312914)

[Figure 1. Mutation rates and phenotype-genotype analysis of the entire cohort (n = 6,043). 7](#_Toc135312915)

[Figure 2. *ASXL1* c.1934dupG-mutated cohort (n = 520) 8](#_Toc135312916)

[Figure 3. *ASXL1* c.1934dupG mutation frequencies and variant allele fractions. 9](#_Toc135312917)

[Figure 4. *ASXL1* mutations in AML by *TP53* and *SETBP1* mutation status. 10](#_Toc135312918)

[Figure 5. *ASXL1* cohesin binding motif-mutated cohort (n = 84) 11](#_Toc135312919)

[References 12](#_Toc135312920)

# **Methods**

Case selection

This retrospective study was performed in adults (≥18 years) with confirmed or suspected myeloid disease who had FoundationOne®Heme testing between January 1, 2014, and August 15, 2021. If a patient had multiple specimens tested at different time points, only the earliest specimen was included. A key limitation was the lack of available comprehensive clinical and laboratory results for review. Thus, we investigated for sampling bias using mutation data. Collectively, 1,105 of the 7,148 patients (16%) that otherwise met inclusion criteria lacked a mutation and were excluded from the study, though it is possible some may have true myeloid neoplasia.

In addition, we only included patients assigned a specific myeloid malignancy diagnosis category, though the lack of available clinical data precluded more definitive evaluation for patients with clonal hematopoiesis of indeterminate potential (CHIP)/clonal cytopenia of undetermined significance (CCUS). Thus, while some included patients may only have CHIP/CCUS, the vast majority of those in our *ASXL1*-mutated cohort of interest had more than one mutation, suggesting clinically relevant myeloid disease.^1^

Sequencing methods and variant calling

FoundationOne®Heme testing was performed in CAP-accredited, CLIA-certified laboratories (Foundation Medicine, Inc., Cambridge, Massachusetts and Morrisville, NC) for all cases. Briefly, bone marrow, peripheral blood, or tissue specimens from patients with known or suspected myeloid neoplasms underwent extraction of DNA and RNA. Libraries were constructed from DNA and cDNA followed by a hybridization-based capture approach for target enrichment as previously described,^2,3^ and pooled libraries were sequenced using the Illumina HiSeq platform. Ancestry^4^ and variant calls were made as previously described,^2,5^ and only those with pathogenic or likely pathogenic functional impacts were included.^6^ Short variants with variant allele fractions (VAF) ≥ 1% were included, with the exception of the *ASXL1* c.1934dupG (p.G646Xfs*12) variant, which was only reported if the VAF was ≥ 15% due to technical limitations of sequencing in this homopolymer site (see Results).^7,8^

Statistical analysis

Fisher’s exact tests and Mann-Whitney U tests were used to evaluate differences between categorical and continuous variables, respectively. Multivariate logistic regression was performed to validate phenotypic distinctions in the cohort and to predict phenotypes based on the presence of co-mutation within the *ASXL1*^mut^ cohort. A two-sided alpha value of 0.05 was considered significant.

# **Table 1. Gene panel**

**Genes with complete exonic coverage.**

| *ABL1* | *ACTB* | *AKT1* | *AKT2* | *AKT3* | *ALK* | *AMER1* | *APC* |
| --- | --- | --- | --- | --- | --- | --- | --- |
| *APH1A* | *AR* | *ARAF* | *ARFRP1* | *ARHGAP26* | *ARID1A* | *ARID2* | *ASMTL* |
| *ASXL1* | *ATM* | *ATRX* | *AURKA* | *AURKB* | *AXIN1* | *AXL* | *B2M* |
| *BAP1* | *BARD1* | *BCL10* | *BCL11B* | *BCL2* | *BCL2L2* | *BCL6* | *BCL7A* |
| *BCOR* | *BCORL1* | *BIRC3* | *BLM* | *BRAF* | *BRCA1* | *BRCA2* | *BRD4* |
| *BRIP1* | *BRSK1* | *BTG2* | *BTK* | *BTLA* | *C11orf30* | *CAD* | *CALR* |
| *CARD11* | *CBFB* | *CBL* | *CCND1* | *CCND2* | *CCND3* | *CCNE1* | *CCNE1* |
| *CCT6B* | *CD22* | *CD274* | *CD36* | *CD58* | *CD70* | *CD79A* | *CD79B* |
| *CDC73* | *CDH1* | *CDK12* | *CDK4* | *CDK6* | *CDK8* | *CDKN1B* | *CDKN2A* |
| *CDKN2B* | *CDKN2C* | *CEBPA* | *CHD2* | *CHEK1* | *CHEK2* | *CIC* | *CIITA* |
| *CKS1B* | *CPS1* | *CREBBP* | *CRKL* | *CRLF2* | *CSF1R* | *CSF3R* | *CTCF* |
| *CTNNA1* | *CTNNB1* | *CUX1* | *CXCR4* | *DAXX* | *DDR2* | *DDX3X* | *DNM2* |
| *DNMT3A* | *DOT1L* | *DTX1* | *DUSP2* | *DUSP9* | *EBF1* | *ECT2L* | *EED* |
| *EGFR* | *ELP2* | *EP300* | *EPHA3* | *EPHA5* | *EPHA7* | *EPHB1* | *ERBB2* |
| *ERBB3* | *ERBB4* | *ERG* | *ESR1* | *ETS1* | *ETV6* | *EXOSC6* | *EZH2* |
| *FAF1* | *FAM46C* | *FANCA* | *FANCC* | *FANCD2* | *FANCE* | *FANCF* | *FANCG* |
| *FANCL* | *FAS* | *FBXO11* | *FBXO31* | *FBXW7* | *FGF10* | *FGF14* | *FGF19* |
| *FGF23* | *FGF3* | *FGF4* | *FGF6* | *FGFR1* | *FGFR2* | *FGFR3* | *FGFR4* |
| *FHIT* | *FLCN* | *FLT1* | *FLT3* | *FLT4* | *FLYWCH1* | *FOXL2* | *FOXO1* |
| *FOXO3* | *FOXP1* | *FRS2* | *GADD45B* | *GATA1* | *GATA2* | *GATA3* | *GID4* |
| *GNA11* | *GNA12* | *GNA13* | *GNAQ* | *GNAS* | *GPR124* | *GRIN2A* | *GSK3B* |
| *GTSE1* | *HDAC1* | *HDAC4* | *HDAC7* | *HGF* | *HIST1H1C* | *HIST1H1D* | *HIST1H1E* |
| *HIST1H2AC* | *HIST1H2AG* | *HIST1H2AL* | *HIST1H2AM* | *HIST1H2BC* | *HIST1H2BJ* | *HIST1H2BK* | *HIST1H2BO* |
| *HIST1H3B* | *HNF1A* | *HRAS* | *HSP90AA1* | *ICK* | *ID3* | *IDH1* | *IDH2* |
| *IGF1R* | *IKBKE* | *IKZF1* | *IKZF2* | *IKZF3* | *IL7R* | *INHBA* | *INPP4B* |
| *INPP5D* | *IRF1* | *IRF4* | *IRF8* | *IRS2* | *JAK1* | *JAK2* | *JAK3* |
| *JARID2* | *JUN* | *KAT6A* | *KDM2B* | *KDM4C* | *KDM5A* | *KDM5C* | *KDM6A* |
| *KDR* | *KEAP1* | *KIT* | *KLHL6* | *KMT2A* | *KMT2C* | *KMT2D* | *KRAS* |
| *LEF1* | *LRP1B* | *LRRK2* | *MAF* | *MAFB* | *MAGED1* | *MALT1* | *MAP2K1* |
| *MAP2K2* | *MAP2K4* | *MAP3K1* | *MAP3K14* | *MAP3K6* | *MAP3K7* | *MAPK1* | *MCL1* |
| *MDM2* | *MDM4* | *MED12* | *MEF2B* | *MEF2C* | *MEN1* | *MET* | *MIB1* |
| *MITF* | *MKI67* | *MLH1* | *MPL* | *MRE11A* | *MSH2* | *MSH3* | *MSH6* |
| *MTOR* | *MUTYH* | *MYC* | *MYCL* | *MYCN* | *MYD88* | *MYO18A* | *NCOR2* |
| *NCSTN* | *NF1* | *NF2* | *NFE2L2* | *NFKBIA* | *NKX2-1* | *NOD1* | *NOTCH1* |
| *NOTCH2* | *NPM1* | *NRAS* | *NSD1* | *NT5C2* | *NTRK1* | *NTRK2* | *NTRK3* |
| *NUP93* | *NUP98* | *P2RY8* | *PAG1* | *PAK3* | *PALB2* | *PASK* | *PAX5* |
| *PBRM1* | *PC* | *PCBP1* | *PCLO* | *PDCD1* | *PDCD11* | *PDCD1LG2* | *PDGFRA* |
| *PDGFRB* | *PDK1* | *PHF6* | *PIK3CA* | *PIK3CG* | *PIK3R1* | *PIK3R2* | *PIM1* |
| *PLCG2* | *POT1* | *PPP2R1A* | *PRDM1* | *PRKAR1A* | *PRKDC* | *PRSS8* | *PTCH1* |
| *PTEN* | *PTPN11* | *PTPN2* | *PTPN6* | *PTPRO* | *RAD21* | *RAD50* | *RAD51* |
| *RAF1* | *RARA* | *RASGEF1A* | *RB1* | *RELN* | *RET* | *RHOA* | *RICTOR* |
| *RNF43* | *ROS1* | *RPTOR* | *RUNX1* | *S1PR2* | *SDHA* | *SDHB* | *SDHC* |
| *SDHD* | *SERP2* | *SETBP1* | *SETD2* | *SF3B1* | *SGK1* | *SMAD2* | *SMAD4* |
| *SMARCA1* | *SMARCA4* | *SMARCB1* | *SMC1A* | *SMC3* | *SMO* | *SOCS1* | *SOCS2* |
| *SOCS3* | *SOX10* | *SOX2* | *SPEN* | *SPOP* | *SRC* | *SRSF2* | *STAG2* |
| *STAT3* | *STAT4* | *STAT5A* | *STAT5B* | *STAT6* | *STK11* | *SUFU* | *SUZ12* |
| *TAF1* | *TBL1XR1* | *TCF3* | *TCL1A* | *TET2* | *TGFBR2* | *TLL2* | *TMEM30A* |
| *TMSB4XP8* | *TNFAIP3* | *TNFRSF11A* | *TNFRSF14* | *TNFRSF17* | *TOP1* | *TP53* | *TP63* |
| *TRAF2* | *TRAF3* | *TRAF5* | *TSC1* | *TSC2* | *TSHR* | *TUSC3* | *TYK2* |
| *U2AF1* | *U2AF2* | *VHL* | *WDR90* | *WHSC1* | *WISP3* | *WT1* | *XBP1* |
| *XPO1* | *YY1AP1* | *ZMYM3* | *ZNF217* | *ZNF24* | *ZNF703* | *ZRSR2* |  |

**Genes with select intronic coverage.**

| *ALK* | *BCL2* | *BCL6* | *BCR* | *BRAF* | *CCND1* | *CRLF2* | *EGFR* |
| --- | --- | --- | --- | --- | --- | --- | --- |
| *EPOR* | *ETV1* | *ETV4* | *ETV5* | *ETV6* | *EWSR1* | *FGFR2* | *IGH* |
| *IGK* | *IGL* | *JAK1* | *JAK2* | *KMT2A* | *MYC* | *NTRK1* | *PDGFRA* |
| *PDGFRB* | *RAF1* | *RARA* | *RET* | *ROS1* | *TMPRSS2* | *TRG* |  |

**Genes with RNA sequencing coverage**

| *ABI1* | *ABL1* | *ABL2* | *ACSL6* | *AFF1* | *AFF4* | *ALK* | *ARHGAP26* |
| --- | --- | --- | --- | --- | --- | --- | --- |
| *ARHGEF12* | *ARID1A* | *ARNT* | *ASXL1* | *ATF1* | *ATG5* | *ATIC* | *BCL10* |
| *BCL11A* | *BCL11B* | *BCL2* | *BCL3* | *BCL6* | *BCL7A* | *BCL9* | *BCOR* |
| *BCR* | *BIRC3* | *BRAF* | *BTG1* | *CAMTA1* | *CARS* | *CBFA2T3* | *CBFB* |
| *CBL* | *CCND1* | *CCND2* | *CCND3* | *CD274* | *CDK6* | *CDX2* | *CHIC2* |
| *CHN1* | *CIC* | *CIITA* | *CLP1* | *CLTC* | *CLTCL1* | *CNTRL* | *COL1A1* |
| *CREB3L1* | *CREB3L2* | *CREBBP* | *CRLF2* | *CSF1* | *CTNNB1* | *DDIT3* | *DDX10* |
| *DDX6* | *DEK* | *DUSP22* | *EGFR* | *EIF4A2* | *ELF4* | *ELL* | *ELN* |
| *EML4* | *EP300* | *EPOR* | *EPS15* | *ERBB2* | *ERG* | *ETS1* | *ETV1* |
| *ETV4* | *ETV5* | *ETV6* | *EWSR1* | *FCGR2B* | *FCRL4* | *FEV* | *FGFR1* |
| *FGFR1OP* | *FGFR2* | *FGFR3* | *FLI1* | *FNBP1* | *FOXO1* | *FOXO3* | *FOXO4* |
| *FOXP1* | *FSTL3* | *FUS* | *GAS7* | *GLI1* | *GMPS* | *GPHN* | *HERPUD1* |
| *HEY1* | *HIP1* | *HIST1H4l* | *HLF* | *HMGA1* | *HMGA2* | *HOXA11* | *HOXA13* |
| *HOXA3* | *HOXA9* | *HOXC11* | *HOXC13* | *HOXD11* | *HOXD13* | *HSP90AA1* | *HSP90AB1* |
| *IGH* | *IGK* | *IGL* | *IKZF1* | *IL21R* | *IL3* | *IRF4* | *ITK* |
| *JAK1* | *JAK2* | *JAK3* | *JAZF1* | *KAT6A* | *KDSR* | *KIF5B* | *KMT2A* |
| *LASP1* | *LCP1* | *LMO1* | *LMO2* | *LPP* | *LYL1* | *MAF* | *MAFB* |
| *MALT1* | *MDS2* | *MECOM* | *MKL1* | *MLF1* | *MLLT1* | *MLLT10* | *MLLT3* |
| *MLLT4* | *MLLT6* | *MN1* | *MNX1* | *MSI2* | *MSN* | *MUC1* | *MYB* |
| *MYC* | *MYH11* | *MYH9* | *NACA* | *NBEAP1* | *NCOA2* | *NDRG1* | *NF1* |
| *NF2* | *NFKB2* | *NIN* | *NOTCH1* | *NPM1* | *NR4A3* | *NSD1* | *NTRK1* |
| *NTRK2* | *NTRK3* | *NUMA1* | *NUP214* | *NUP98* | *NUTM2A* | *OMD* | *P2RY8* |
| *PAFAH1B2* | *PAX3* | *PAX5* | *PAX7* | *PBX1* | *PCM1* | *PCSK7* | *PDCD1LG2* |
| *PDE4DIP* | *PDGFB* | *PDGFRA* | *PDGFRB* | *PER1* | *PHF1* | *PICALM* | *PIM1* |
| *PLAG1* | *PML* | *POU2AF1* | *PPP1CB* | *PRDM1* | *PRDM16* | *PRRX1* | *PSIP1* |
| *PTCH1* | *PTK7* | *RABEP1* | *RAF1* | *RALGDS* | *RAP1GDS1* | *RARA* | *RBM15* |
| *RET* | *RHOH* | *RNF213* | *ROS1* | *RPL22* | *RPN1* | *RUNX1* | *RUNX1T1* |
| *RUNX2* | *SEC31A* | *SEPT5* | *SEPT6* | *SEPT9* | *SET* | *SH3GL1* | *SLC1A2* |
| *SNX29* | *SRSF3* | *SS18* | *SSX1* | *SSX2* | *SSX4* | *STAT6* | *STL* |
| *SYK* | *TAF15* | *TAL1* | *TAL2* | *TBL1XR1* | *TCF3* | *TCL1A* | *TEC* |
| *TET1* | *TFE3* | *TFG* | *TFPT* | *TFRC* | *TLX1* | *TLX3* | *TMPRSS2* |
| *TNFRSF11A* | *TOP1* | *TP63* | *TPM3* | *TPM4* | *TRIM24* | *TRIP11* | *TTL* |
| *TYK2* | *USP6* | *WHSC1* | *WHSC1L1* | *YPEL5* | *ZBTB16* | *ZMYM2* | *ZNF384* |
| *ZNF521* |  |  |  |  |  |  |  |

# **Table 2. Patient cohort**

| **Feature** | **AML** | **MDS** | **MDS/MPN** | **MPN** | **Total** |
| --- | --- | --- | --- | --- | --- |
| Total patients | 2,214 (37%) | 2,413 (40%) | 363 (6.0%) | 1,053 (17%) | 6,043 |
| *ASXL1*^mut^ | 408 (18%) | 530 (22%) | 193 (53%) | 283 (27%) | 1,414 (23%) |
| c.1934dupG (p.G646Wfs) | 160 (39%) | 172 (32%) | 82 (42%) | 106 (37%) | 520 (37%) |
| Others | 248 (61%) | 358 (68%) | 111 (58%) | 177 (63%) | 894 (63%) |
| c.1900_1922del (p.E635Rfs) | 60 (15%) | 77 (15%) | 23 (12%) | 31 (11%) | 191 (14%) |
| c.2077C>T (p.R693*) | 21 (5.1%) | 26 (4.9%) | 6 (3.1%) | 13 (4.6%) | 66 (4.7%) |
| Cohesin-binding motif | 23 (5.6%) | 34 (6.2%) | 16 (8.3%) | 11 (3.9%) | 84 (5.9%) |
| Median age (IQR) | 64 (51-73) | 71 (63-79) | 71 (64-78) | 67 (55-75) | 68 (58-76) |
| *ASXL1*^wt^ | 62 (48-72) | 71 (61-78) | 71 (63-78) | 64 (52-73) | 67 (55-75) |
| *ASXL1*^mut^ | 70 (63-77) | 74 (66-79) | 71 (65-77.5) | 71 (65-78) | 72 (65-78) |
| P value (*ASXL1*^mut^ vs. *ASXL1*^wt^) | <0.0001 | <0.0001 | 0.3995 | <0.0001 | <0.0001 |
| Male:female | 1.34:1 | 1.52:1 | 1.81:1 | 1.18:1 | 1.40:1 |
| *ASXL1*^wt^ | 1.21:1 | 1.36:1 | 1.66:1 | 0.97:1 | 1.23:1 |
| *ASXL1*^mut^ | 2.19:1 | 2.35:1 | 1.97:1 | 2.08:1 | 2.19:1 |
| P value (*ASXL1*^mut^ vs. *ASXL1*^wt^) | <0.0001 | <0.0001 | 0.4437 | <0.0001 | <0.0001 |
| European:non-European ancestry | 3.08:1 | 4.02:1 | 5.26:1 | 3.26:1 | 3.54:1 |
| *ASXL1*^wt^ | 2.97:1 | 3.82:1 | 5.54:1 | 3.03:1 | 3.36:1 |
| *ASXL1*^mut^ | 3.64:1 | 4.89:1 | 5.03:1 | 4.05:1 | 4.32:1 |
| P value (*ASXL1*^mut^ vs. *ASXL1*^wt^) | 0.1268 | 0.0564 | 0.7754 | 0.1007 | 0.0009 |
| Mutations (median, IQR) | 4 (2-5) | 2 (1-4) | 4 (3-5) | 2 (1-4) | 3 (2-4) |
| *ASXL1*^wt^ | 3 (2-5) | 2 (1-3) | 3 (2-4) | 2 (1-3) | 2 (1-4) |
| *ASXL1*^mut^ † | 4 (3-5) | 3 (2-4) | 4 (2-5) | 3 (2-4) | 3 (2-5) |
| P value (*ASXL1*^mut^ vs. *ASXL1*^wt^) | <0.0001 | <0.0001 | 0.0003 | <0.0001 | <0.0001 |

† Excludes *ASXL1* mutations.

AML: acute myeloid leukemia; MDS: myelodysplastic syndrome; MPN: myeloproliferative neoplasm; IQR: interquartile range

# **Figure 1. Mutation rates and phenotype-genotype analysis of the entire cohort (n = 6,043).**

**
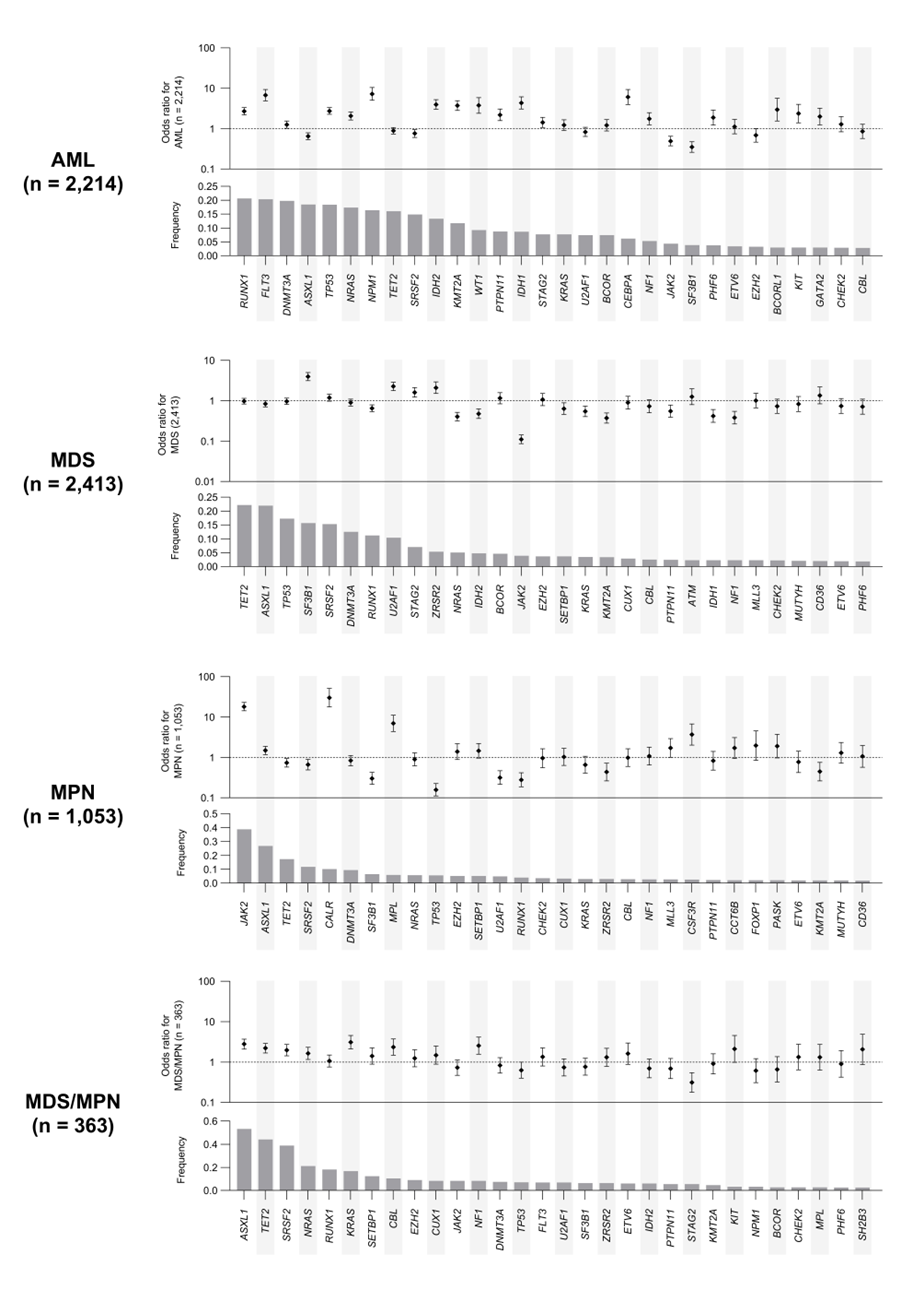
**

#
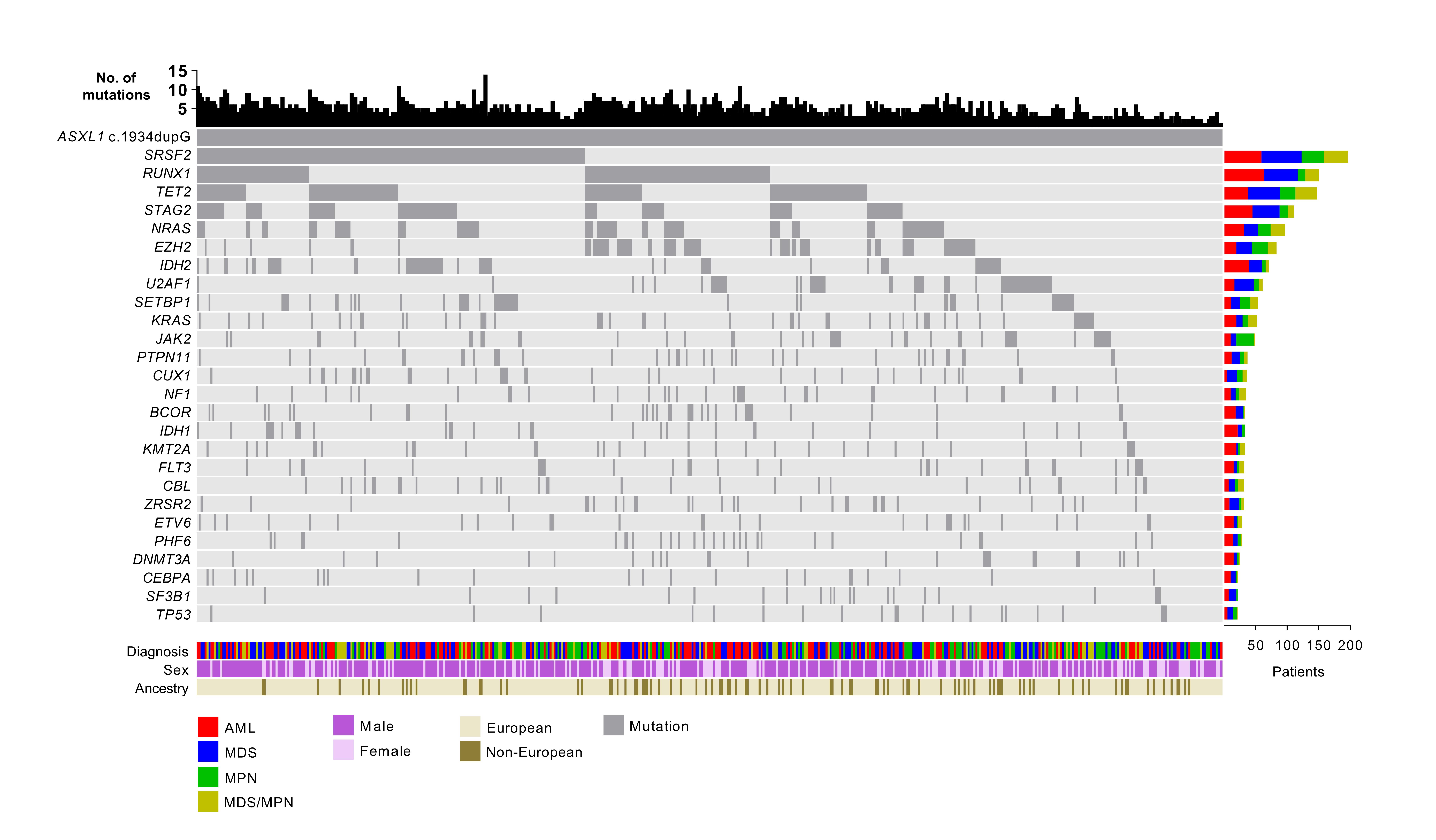
**Figure 2. *ASXL1* c.1934dupG-mutated cohort (n = 520)**

The top 25 mutated genes in the cohort are represented.

# **Figure 3. *ASXL1* c.1934dupG mutation frequencies and variant allele fractions.**

**
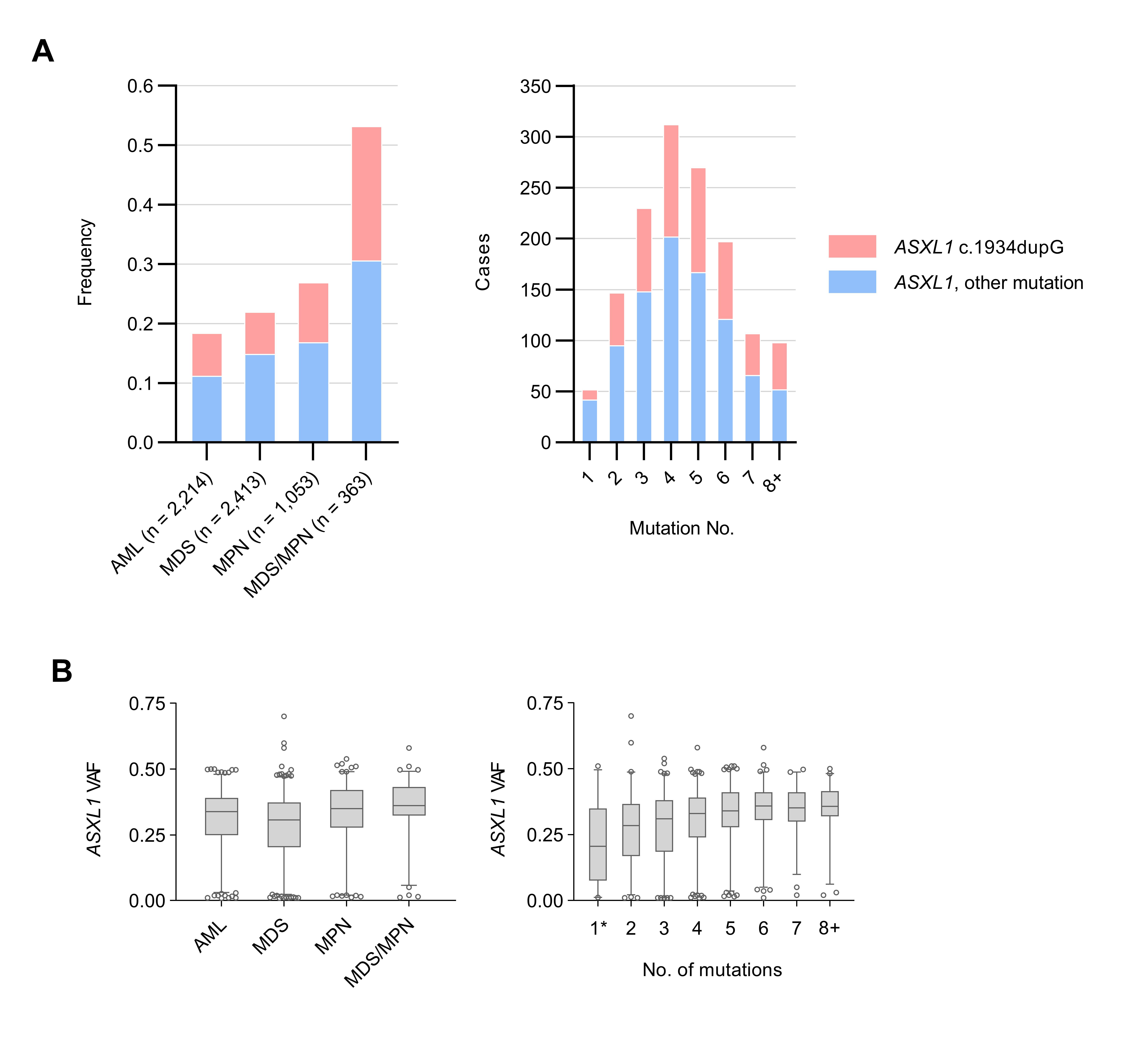
**

* Patient’s sole mutation was in *ASXL1.*

#
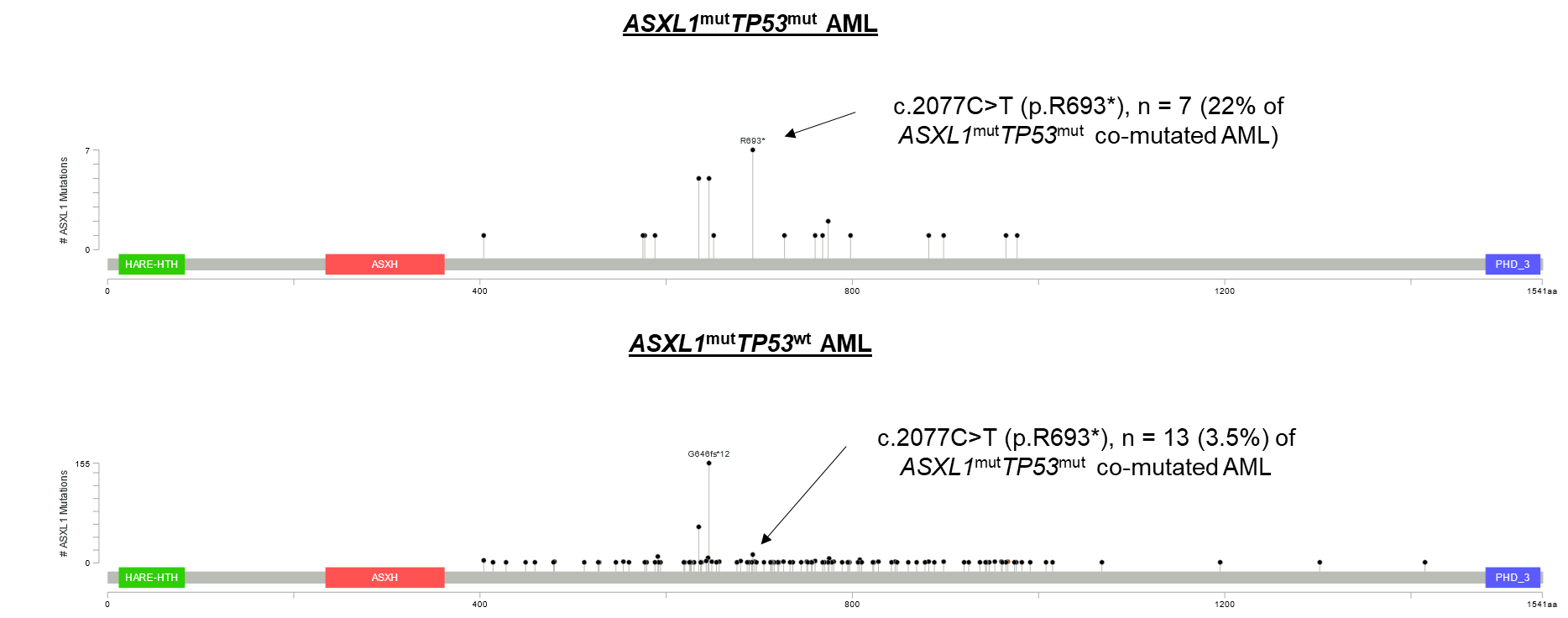
**Figure 4. *ASXL1* mutations in AML by *TP53* and *SETBP1* mutation status.**

^wt^


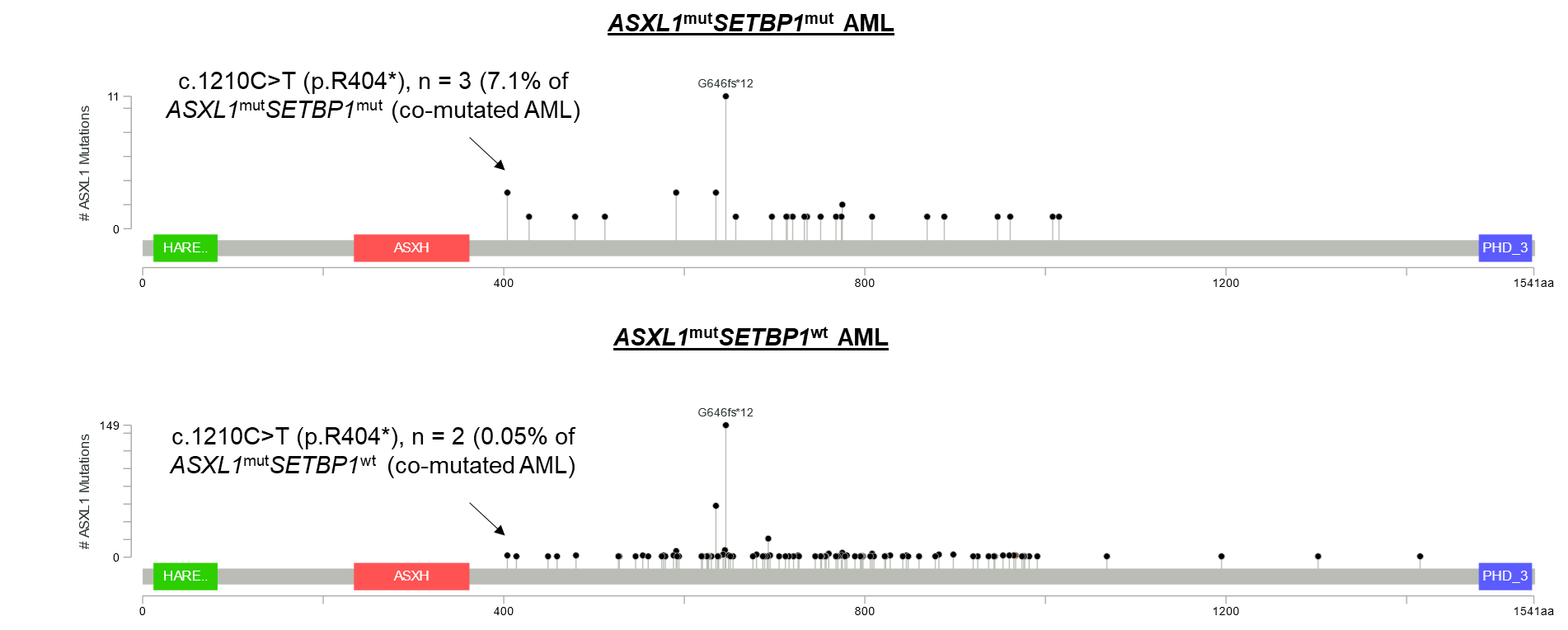


Figure generated using the mutation mapper feature in cBioPortal.^9,10^

# **
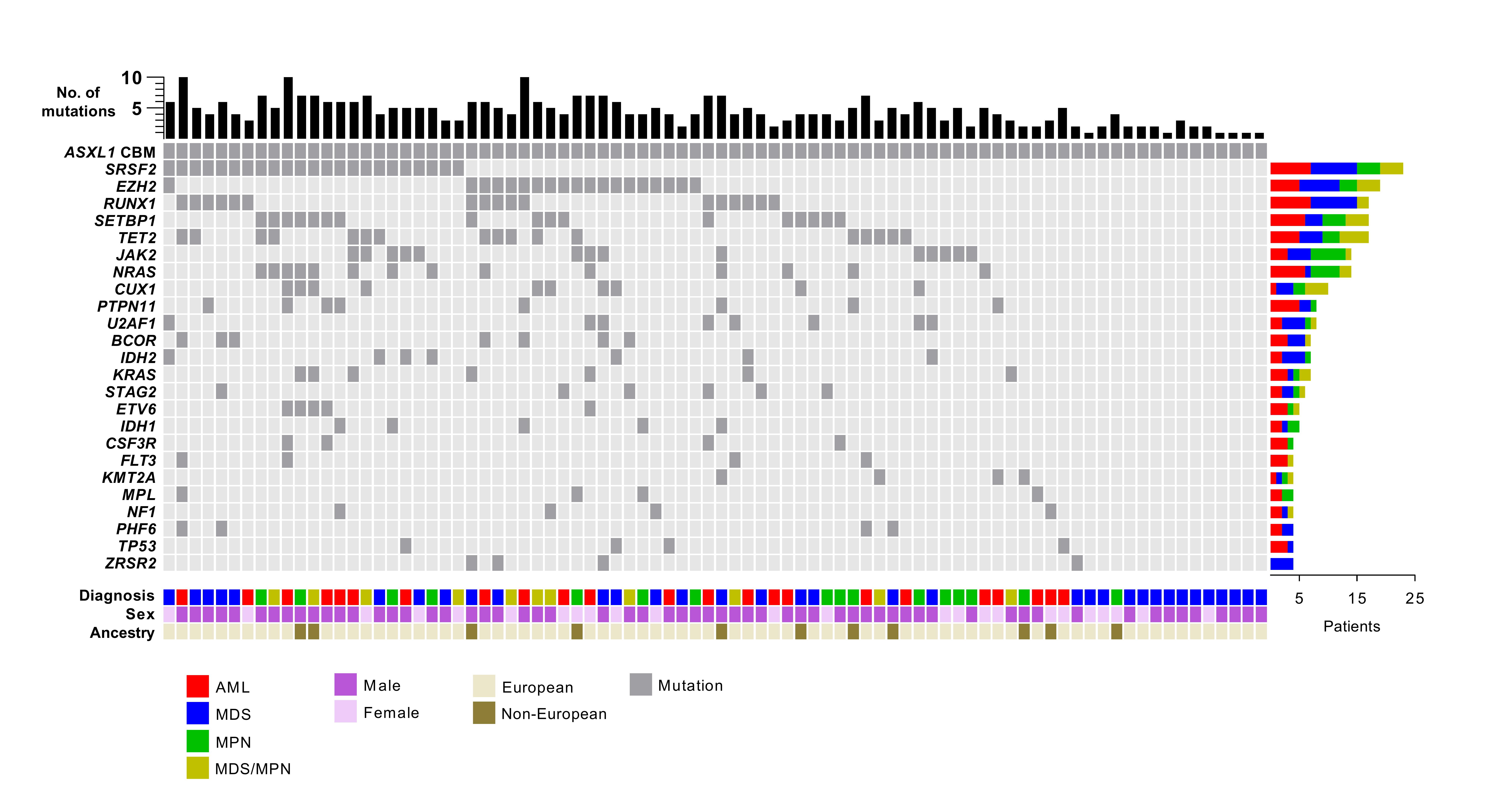
Figure 5. *ASXL1* cohesin binding motif-mutated cohort (n = 84)**

The top 25 mutated genes in the cohort are represented.

# **References**

1. Malcovati L, Gallì A, Travaglino E, et al. Clinical significance of somatic mutation in unexplained blood cytopenia. *Blood*. 2017;129(25):3371–3378.

2. He J, Abdel-Wahab O, Nahas MK, et al. Integrated genomic DNA/RNA profiling of hematologic malignancies in the clinical setting. *Blood*. 2016;127(24):3004–3014.

3. Tarlock K, Zhong S, He Y, et al. Distinct age-associated molecular profiles in acute myeloid leukemia defined by comprehensive clinical genomic profiling. *Oncotarget*. 2018;9(41):26417–26430.

4. Newberg J, Connelly C FG. Determining patient ancestry based on targeted tumor comprehensive genomic profiling [abstract]. *Proc. Am. Assoc. Cancer Res. Annu. Meet. 2019 Suppl)Abstract nr 1599.* .

5. Frampton GM, Fichtenholtz A, Otto GA, et al. Development and validation of a clinical cancer genomic profiling test based on massively parallel DNA sequencing. *Nat. Biotechnol.* 2013;31(11):1023–1031.

6. Li MM, Datto M, Duncavage EJ, et al. Standards and Guidelines for the Interpretation and Reporting of Sequence Variants in Cancer: A Joint Consensus Recommendation of the Association for Molecular Pathology, American Society of Clinical Oncology, and College of American Pathologists. *J. Mol. Diagnostics*. 2017;19(1):4–23.

7. Yannakou CK, Jones K, McBean M, et al. ASXL1 c.1934dup;p.Gly646Trpfs*12—a true somatic alteration requiring a new approach. *Blood Cancer J.* 2017;7(12):656.

8. Montes-Moreno S, Routbort MJ, Lohman EJ, et al. Clinical molecular testing for ASXL1 c.1934dupG p.Gly646fs mutation in hematologic neoplasms in the NGS era. *PLoS One*. 2018;13(9):.

9. Cerami E, Gao J, Dogrusoz U, et al. The cBio Cancer Genomics Portal: An open platform for exploring multidimensional cancer genomics data. *Cancer Discov.* 2012;2(5):401–404.

10. Gao J, Aksoy BA, Dogrusoz U, et al. Integrative analysis of complex cancer genomics and clinical profiles using the cBioPortal. *Sci. Signal.* 2013;6(269):.
